# Developing an interior cladding fiberboard by utilizing sugarcane bagasse as a local agro-waste in Egypt

**DOI:** 10.1038/s41598-023-39860-6

**Published:** 2023-08-08

**Authors:** Marianne Nabil Guirguis, Zainab Farahat, Amany Micheal

**Affiliations:** 1 Architectural Engineering Department, Faculty of Engineering, The British University in Egypt, El-Sherouk City, Egypt; 2 Centre for Advanced Materials (CAM), The British University in Egypt, El-Sherouk City, Egypt

**Keywords:** Engineering, Materials science

## Abstract

The conception of materials with fewer carbon dioxide emissions, using natural fibers, and recycling resources, is of increasing relevance to the world today to combat climatic change and pollution. This is a significant step toward reducing the environmental effect of building materials and addressing a multitude of sustainable development goals (SDGs) in a direct or indirect way. This research investigates using sugarcane bagasse (SCB) as a local green base material in Egypt for creating composite fiberboard that can be used in a multitude of architectural applications as an interior cladding board and was found to have thermal insulation qualities, achieving a dual aim of aesthetically pleasing interiors, in addition to a step towards thermal comfort, thus, enhancing human well-being. At the same time, this will cut down on energy use and carbon emissions. Finally, creating a partially green cladding particleboard will decrease the environmental impact two-fold, utilizing abundant agro-waste and hence, eliminating its disposal hazards, and simultaneously decreasing the environmental impact of construction material in its life cycle. Relevant mechanical and physical properties of the developed board were experimentally tested to investigate and characterize its material, hence, validate its potential operability.

## Introduction

Climate change is one of the most important issues facing the entire world in the twenty-first century^1^. It has become increasingly debated in terms of actions to be taken in order to limit its genuine predictions and propose solution for the problems which are facing humanity, rather than as a dispute over the scientific phenomenon or its impact, in order to avoid the significant alterations in the external environment caused by climatic conditions. The most substantial energy consumer and greenhouse gas emitter annually is buildings^2^. The building and construction industry consumes huge resources and emits a lot of pollution globally. The ability of a technology to be sustained over the long term has been one of the most important factors in judging its viability in recent years. Any new technique needs to be created with environmentally friendly practices in mind, including the use of non-toxic and organic solvents, the discharge of no hazardous materials into the environment, and minimal energy consumption. One of the most popular methods to reduce manufacturing costs is to use current wastes and transform them into high-value engineered goods.

Additionally, technologies for producing renewable items from waste must be fully developed for long-term sustainability^3^. Spiegle asserts that employing environmentally sustainable green building materials helps to meet the market's growing need for natural, non-toxic, energy-efficient, and environmentally friendly products^4^. Green building materials assure the sustainable use of the planet's resources. Hey interact within the confines of natural cycle and ecosystem interactions. In green construction, nontoxic materials are used, they’re made of recycled materials and sometimes can be reused as well. They are both energy and water efficient. Green products are those that are environmentally friendly in their production, use, and recycling processes. Sustainable green materials have a positive impact on resource management, waste management and (IEQ) indoor environmental quality, and efficiency^5^.

Hence, this research aims to create a composite fiberboard that can be utilized in the building industry as a particleboard with by using one of the most abundant local agro-waste material fiber in Egypt; SCB. The board is fabricated. Its properties are then investigated through an experimental approach, by characterizing the board’s physical and mechanical properties. Previous trials of developing and characterizing different composite materials were spotted; some of which applied binders such as Polyvinyl alcohol (PVA)^6^, foam^7^, starch, formaldehyde resin with hardener emulsified paraffin wax, polyurethane, or epoxy^6,8–11^. The proposed fiberboard utilizes sugarcane bagasse as a base material utilizing one of the most commonly used binders in the building industry; epoxy resin as a binding matrix. The originality of the research is that the developed board was characterized to determine its proposed industrial usage as an interior cladding board, with the most relevant mechanical and physical properties and compared to other boards to understand where it stands comparatively in relation to other previously developed materials.

## Literature review on sugarcane bagasse

Sugarcane Bagasse is a bio-product of sugarcane fiber which is retained after the juice has been extracted from the sugarcane^12^, resultant from the squeezing and crushing process in the form of a fibrous residue^13^. SCB waste is chosen as an ideal raw material in manufacturing new products because of its low fabricating costs and high quality green end material. It is a suitable green material since it is widely available due to widespread sugarcane planting in many locations around the world^14,15^, providing a valid and consistent supply^16^. Hence, it is an easy to obtain accessible and sustainable material^17^.

In Egypt, sugarcane is an abundant crop cultivated mainly in Upper Egypt^18^ (the land surrounding the southern part of the River Nile in Egypt till Sudan)^14^. Its main components are of cellulose, hemicellulose and lignin, in addition to ash and some extractives^19,20^. The main components of the SCB are listed in Table 1

**Table 1:** Composition of bagasse^21^ (Rabelo, 2015).

Component	Composition (%)
Glucose	19.5
Xylose	10.5
Arabinose	1.5
Galactose	0.55
Lignin	9.91
Organo soluble	2.7
Reducing sugars	1.85
Uronic acids	1.91
Ash	1.6
Cellulose	50
Total hexoses	20.04
Total pentoses	12

The main purposes for sugarcane harvesting locally in Egypt are sugar and molasses production, in addition to seeds for subsequent cultivation and juice. The largest of which is the first. Sugarcane milling process results in many products and byproducts^22^ such as such as green tops, dry leaves, molasses, soil, mud, furnace ash and SCB^23^. According to research, SCB presents 30% of the cultivated sugarcane as an agriculture crop^24^. In 2020, the amount of cultivated sugarcane in Egypt was 14.9 million tons^25^. Thus, the amount of SCB can be deduced by calculations is about 4.5 million tons. About 30% of this amount is lost due to uncontrolled burning, causing pollution and presenting a lost opportunity for a recyclable, biodegradable material^23^. As it is considered one of the largest agro-waste materials in Egypt^26^. Recycling it is the optimum eco-friendly solution that reduces the negative effect of burning^27^. This open burning process contributes to environmental issues such as air pollution, degraded soil, and global warming^28^.

### Sugarcane bagasse thermal properties

According to a recent study, sugarcane bagasse as a plant fiber with density: 100–125 kg/m^3^ had thermal conductivity 0.046–0.049 W/(m K) which is lower than coconut husk, sunflower fiber, cotton stalk fiber, coriander stalk fiber and date palm fiber^29^. Meherzad at al.^30^ tested a group of specimens with thickness 20, 30, and 40 mm and unit weight 100, 150, and 200 kg/m^3^. The used resin is Polyvinyl alcohol water-soluble polymer. The thermal conductivity of the fabricated specimens ranges between 0.00157 and 0.0021 W/(m k) for specimens of thickness 20mm and three densities 100–200 kg/m^3^. The other specimens with thickness 30mm, the thermal conductivity ranges between 0.00189 and 0.00177 W/(m k) for the same densities. For specimens with thickness 40 mm, the corresponding values are 0.00146 to 0.00203 W/(m k). The Thermal properties of composites of cement and SCB can be found in the work of Onésippe *et al*. (2010). According to Abedom^9^, composite materials with a mix of Bagasse/Bamboo fibers exhibited thermal conductivity values between 0.130 and 0.086 W/(m K) for percentage 100%, 70%, 50%, 30%, and 0% of bagasse. Previous research investigated the effect of the amount of fiber on the thermal behavior of composites made of cement composites reinforced with vegetable bagasse fibers; a decrease in thermal conductivity from 0.62 to 0.46 W/(m K) was encountered in composites when the content of fiber was increased from 1.5 to 3.0%, respectively^31^. Also, for light particleboard from sugarcane bagasse residues, the thermal conductivity varied from (0.045 to 0.0051 W/(m K), which is in the range of other insulation boards made of ligno-cellulosic materials^32^. Researchers discovered that ceiling insulation boards made completely of sugarcane bagasse with a 470.3 kg/m^3^ density had a better thermal conductivity than other components frequently used in the ceilings such as Plaster of Paris, asbestos, and PVC^33^. According to^29^, thermal conductivity is influenced by fiber composition. The efficiency of thermal insulating materials is highly depending on the thermal conductivity (K) of the materials which depends on various factors such as density, porosity and Moisture content of the material^34^.

### Sugarcane bagasse physical properties

Research has been conducted to determine the density, absorbency, and dimensional constancy of polymeric combinations made of bagasse fibers. In general, natural fibers demonstrate low density with relatively high toughness, thus, exhibit acceptable mechanical properties^35^. According to Ramlee, the effect of soaking bagasse fiber-based particle board in heated and cool water for various durations on its mechanical properties was investigated. The mechanical properties of the combination were severely reduced by both hot and cold-water submersion. In addition, as compared to unprocessed bagasse fiber-based combinations, poly (methylene polyphenyl isocyanate) treated particle boards showed good damp resistance^29^.

Previous research revealed the potential of adding SCB^36^ as a reinforcement due to its mechanical features including flexural hardness, tensile strength, flexural absolute value, stiffness, and impact strength when suitable adjustments and production technologies are used^16,20^. Previous research of particleboards with different percentages of bagasse determined that its use for particleboard fabrication resulted in enhanced water absorption and thickness swelling characteristics^37^. It can be chemically treated and modified, as well as combining well with different binding materials to develop various forms of the new compound materials^16^.

## Experimental work

**Figure 1:**
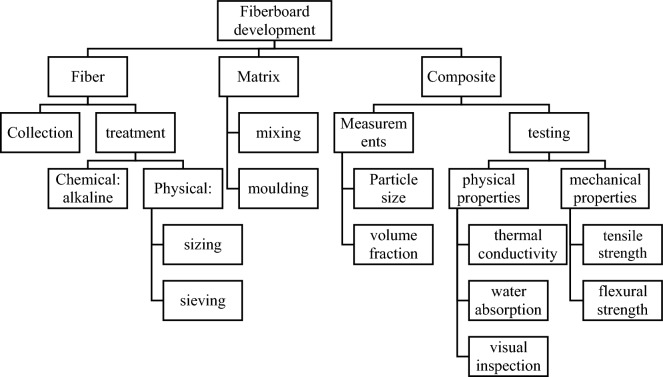
Fiberboard development process method.

The purpose of the experiment is to develop a composite panel board with potentially good thermal insulation properties by utilizing SCB as the base material fiber and applying epoxy resin as the binding matrix. The methodology adopted in the experimental procedures is illustrated in Fig. 1.

### SCB treatment

**Figure 2:**
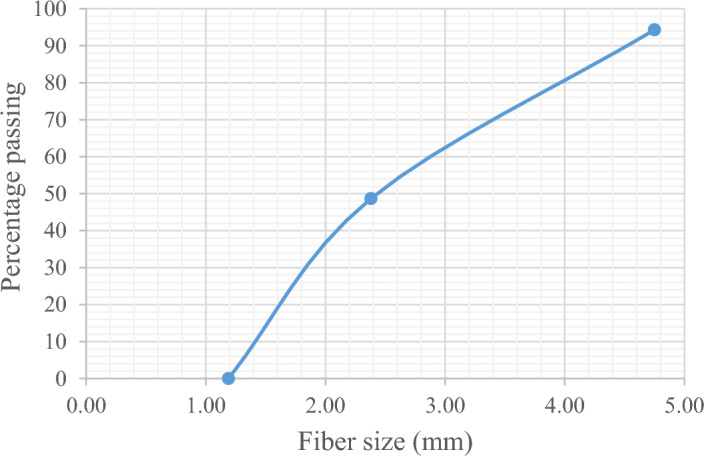
Fiber size distribution curve (by authors).

SCB fibers should be treated before incorporating them in composite manufacturing^12^. The chemical treatment aims to enhance resistance against water and to improve compatibility with polymeric matrix. Different regimens of treatment can be found in the literature^12,38,39^. The adopted technique of treatment in this research is by washing the fiber with warm water several times to remove the sugar. Afterwards, the bagasse fibers are cut to smaller particles for sizing. Then, chemical treatment takes place using Sodium Hydroxide NaOH, by drenching the fibers in a 1:10 concentration Sodium Hydroxide solution for 3 h, and then the fibers are washed with water to remove the solution. The fibers are left to dry in an oven to reduce water content at a temperature of 150 °C. After this treatment process, the fibers color changes to light yellow. To identify the density of the bagasse fibers, the pycnometer is used. The treated SCB fibers density ρ is 390 kg/m^3^. A sieve analysis test is conducted on the dry sample and the particle size distribution curve is shown in Fig. 2. The particle size ranges between 1.2 and 5 mm.

### Specimens' manufacturing

**Figure 3:**
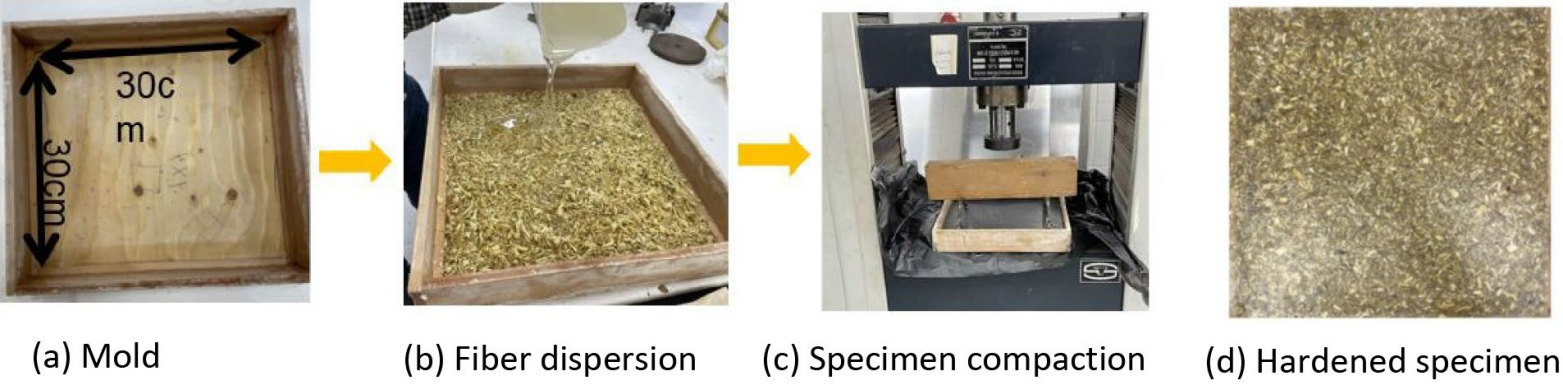
Specimen manufacturing process (by authors).

Different trials are conducted to obtain a specimen with minimum thickness and highest fiber volume fraction. The manufacturing and testing take place in the Centre for Advanced Materials (CAM) hosted by the British University in Egypt. The adopted technique is to distribute the fibers in a 300 × 300 mm mould then to pour the resin. The matrix used in preparing all composite specimens is Araldite LY1564 epoxy resin mixed with Aradur 3486 hardener at weight ratio of 2:1. Huntsman Advanced Materials, Switzerland, supplies both components. The density of the resin matrix is 1.178 gm/cm^3^. To ensure good compaction of the specimen, the specimen is placed under a compression load of 12.8 kN for 24 h as shown in Fig. 3.

The specimen is then removed from the mold and the thickness is measured. The specimen is 9 mm thick and with good appearance with uniformly distributed fibers. Volume Fraction Equation is used to evaluate the fiber volume fraction of the composite.

1 $$cf=\frac{\rho m*Wf}{\rho m*Wf+\rho fWm}$$

where $cf$ = fiber volume fraction, $\rho m$ = density of matrix, $Wf$ = weight of fiber in the sample, and, $\rho f$ = density of fiber, $Wm$=weight of matrix. 

According to Eq. (1), the fiber volume fraction is 39%.

### Physical properties for the specimen

**Figure 4:**
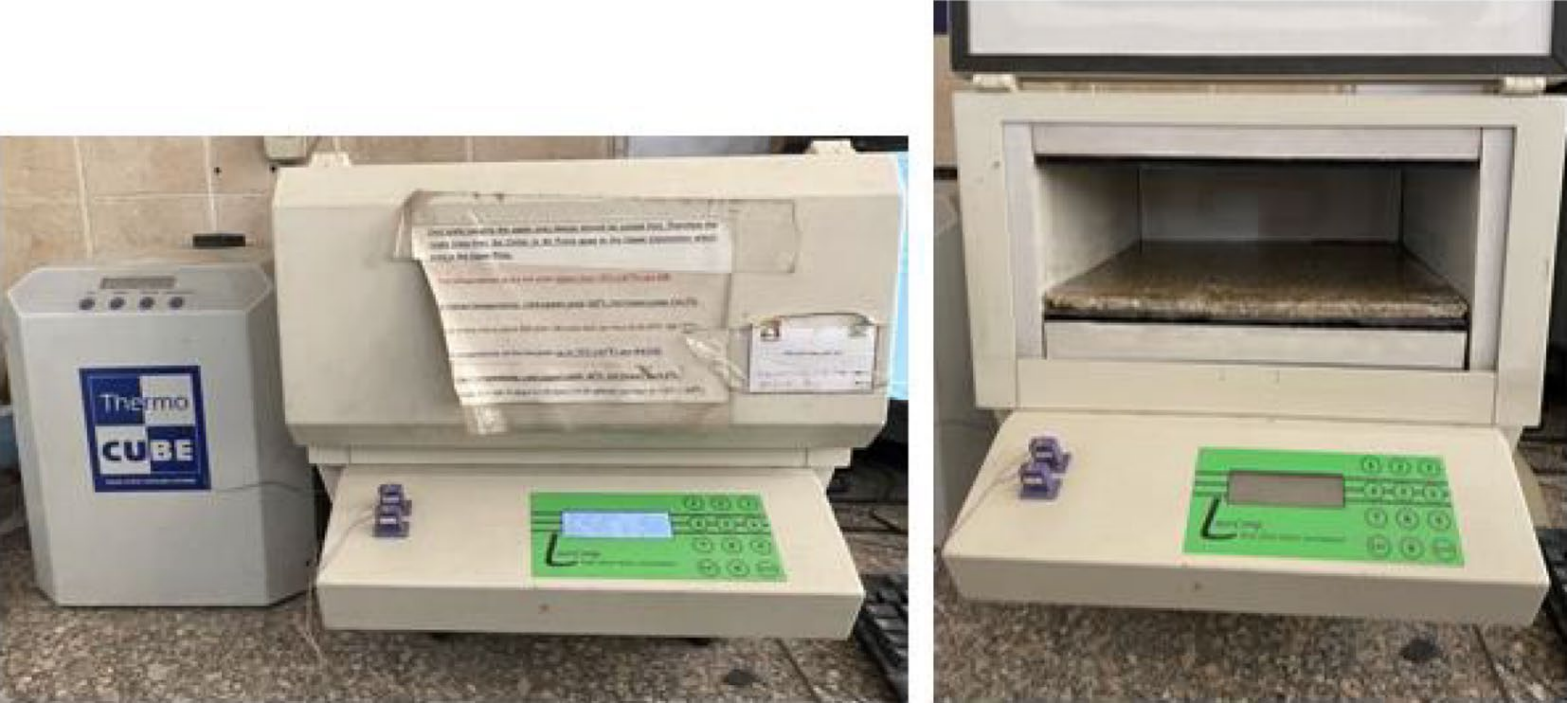
Thermal conductivity test using LaserComp Heat Flow meter (by authors).

The physical properties measured are the density, absorption and thermal conductivity. The procedures to determine the density of the board are according to ASTM-D2395. The density ρ is found to be is 1.08 gm/cm^3^. The thermal conductivity test is conducted to test the ability of the material to transmit heat measured in W/(m k). This test is done at The Housing and Building Research Centre (HBRC) in Cairo using LaserComp Heat Flow Meter instrument. The Heat Flow Meter method, designed specifically for insulating materials, is defined by ASTM C518-17^40^, ISO 8301:1991^41^, and DIN EN 12667 (2001)^42^. This cost-effective and practical method for this sample size (300 mm × 300 mm × 9 mm) and is widely recognized and preferred by industry professionals throughout the world for its speed, and simplicity^43^. The thermal conductivity test was conducted according to the standard specification ASTM C-518, with environmental temperature of 24C°. The coefficient of thermal conductivity of the sample is 0.093 W/(m K). The experimental setting is shown in Fig. 4.

**Figure 5:**
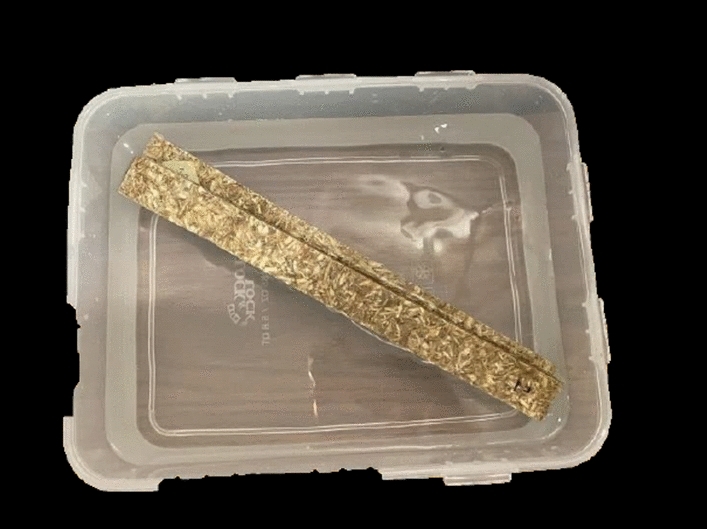
Absorption test by soaking the samples in water for 24 Hrs (by authors).

The third physical property measured for the board is the absorption. The test is performed according to ASTM C1763-20^44^. This test was done using sample strips with the dimensions of 250 × 25 × 9 mm as per Fig. 5 and under ambient temperature of 23 °C.

**Figure 6:**
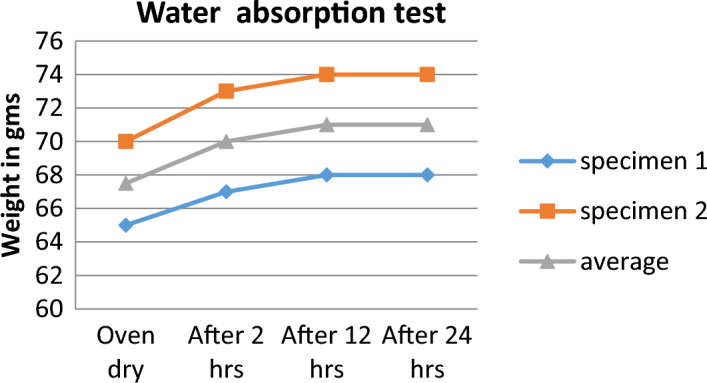
Graph to illustrate water absorption test for 24 h (by authors).

The samples are weighed in the over dry condition, then, they are soaked in water at 21 C^o^. The samples are weighed after 2 h, 12 h, and 24 h to ensure moisture equilibrium. The test results are presented in Table 2 and at 24 h soaking the moisture equilibrium stage is reached. The average weight of the oven-dry samples and saturated samples are 67.5 gm and 71 gm respc. Figure 6 presents the water absorption test results for 24 h. The board absorption can be calculated using Eq. (2): Absorption Equation.

**Table 2:** Water absorption test results (by authors).

Specimen number	Specimen weight			
	Oven dry	Soaking for 2 h	Soaking for 12 h	Soaking for 24 h
1	65	67	68	68
2	70	73	74	74
Average	67.5	70	71	71

2 $$M\%=\frac{Wi-Wo}{Wo}\times 100$$

where: M = absorption %, Wi = current specimen mass, g, and, Wo = oven-dry specimen mass, g

According to Eq. (2), the absorption is 5.18%.

### Mechanical properties

The mechanical properties of the manufactured board are with significance to ensure its capacity to installation or any other architectural application. The mechanical properties of concern in this work are the tensile strength and the flexural strength. The conducted tests are in compliance with ASTM D3039^45^ and ASTM D7264^46^ for tensile and flexural strength respectively.

### Tensile strength test for the specimen

**Figure 7:**
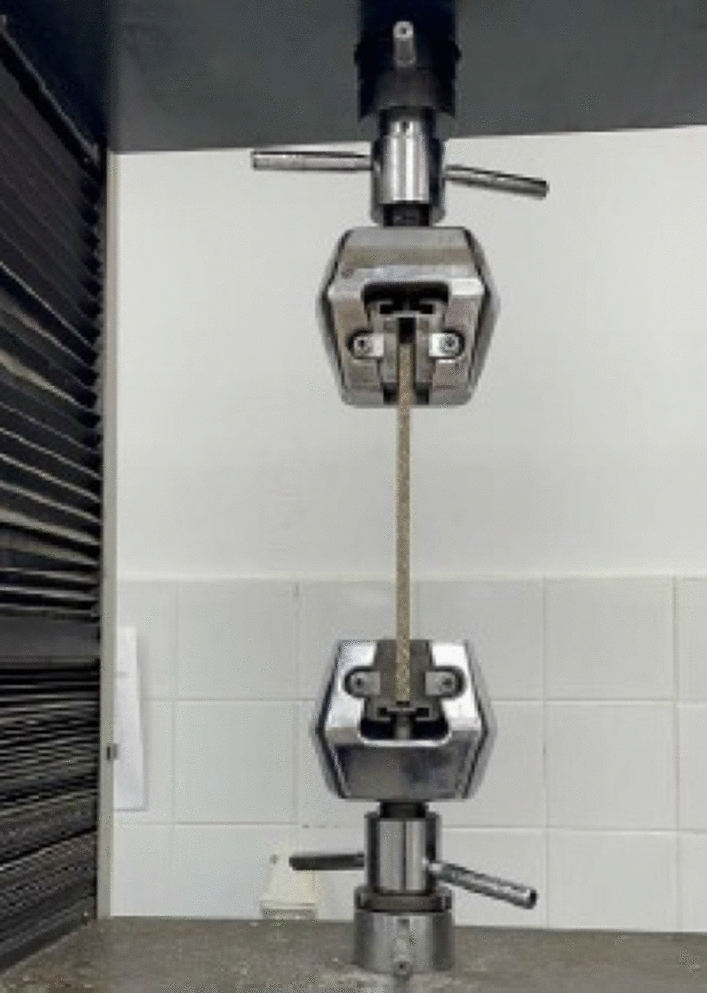
Tensile strength test setup (by authors).

**Figure 8:**
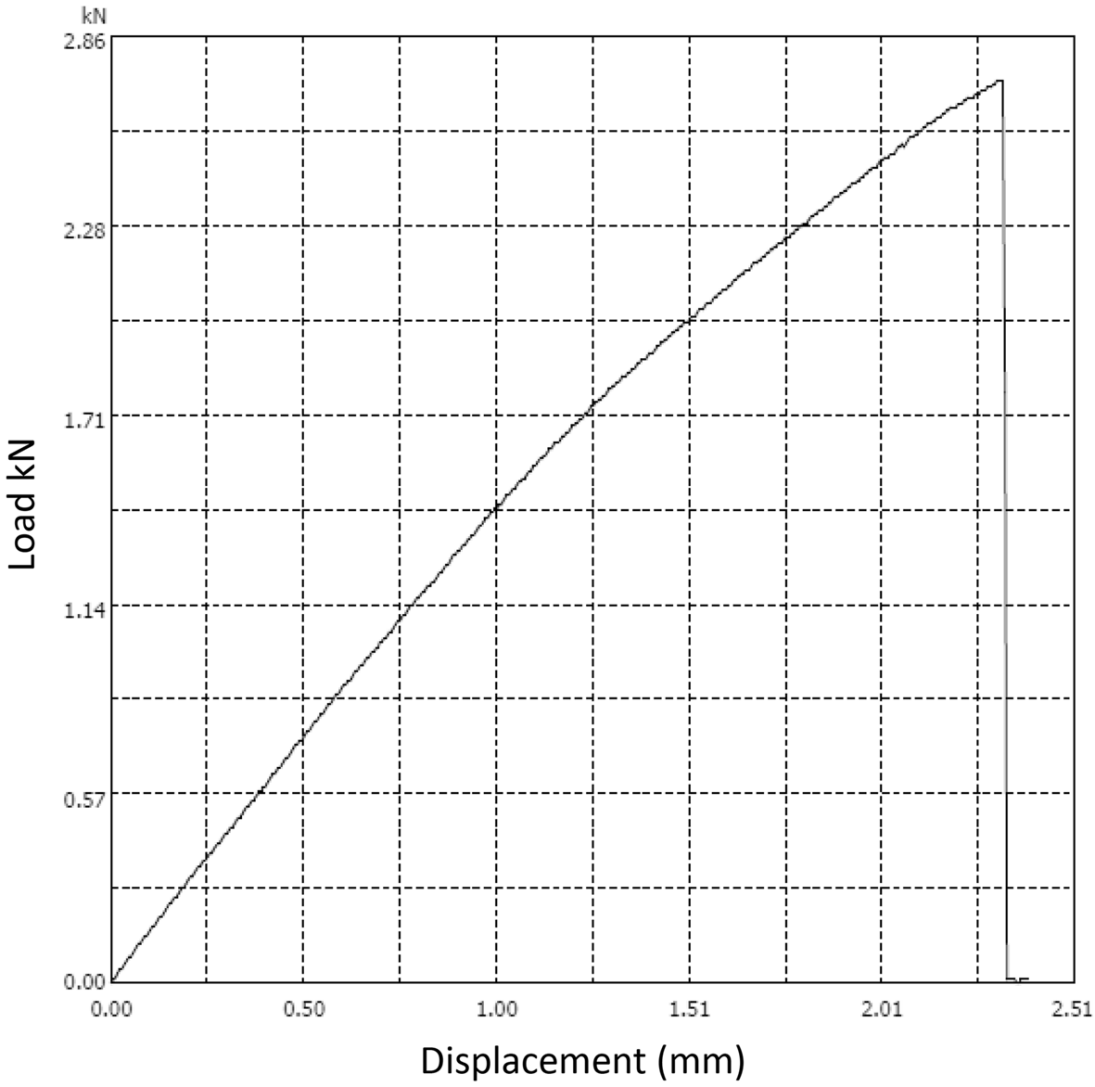
Load deformation curve (by authors).

For tensile strength, the specimens are of dimensions 250 × 25 × 9 mm, the gauge length is 200 mm. Four specimens are tested. The tensile testing machine is Microcomputer Controlled Electronic Universal Testing Machine model WDW-100D. The machine is displacement control, and the loading rate is 2 mm/min for tensile strength test according to ASTM D3039. The test set up is shown in Figs. 7. The load-deformation of the specimen is shown in Fig. 8. The specimen number, relevant strength and stiffness, average strength, average stiffness, and the standard deviation of the results are shown in Table 3.

**Table 3:** Tensile strength test results.

Specimen number	Tensile strength (MPa)	Module of elasticity (MoE) GPa	Average strength (MPa)	Average stiffness (MoE) (GPa)	Standard Deviation of the strength (MPa)	Standard Deviation of the MoE (GPa)
1	13	1.17	12	1.23	1.41	0.0711
2	13	1.31				
3	12	1.27				
4	10	1.17				

### Flexural strength test for the specimen

**Figure 9:**
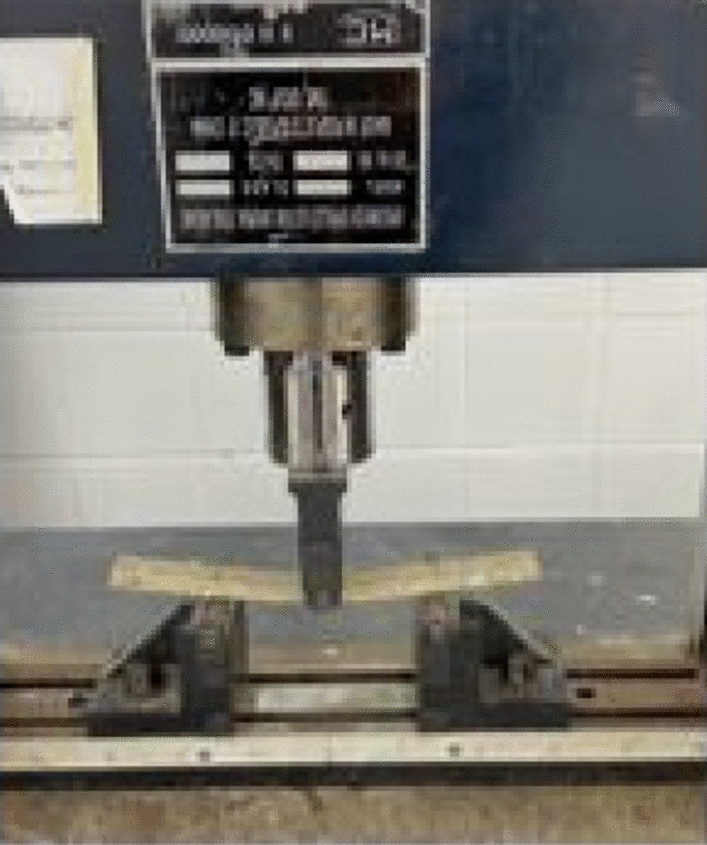
Flexural strength test setup (by authors).

**Figure 10:**
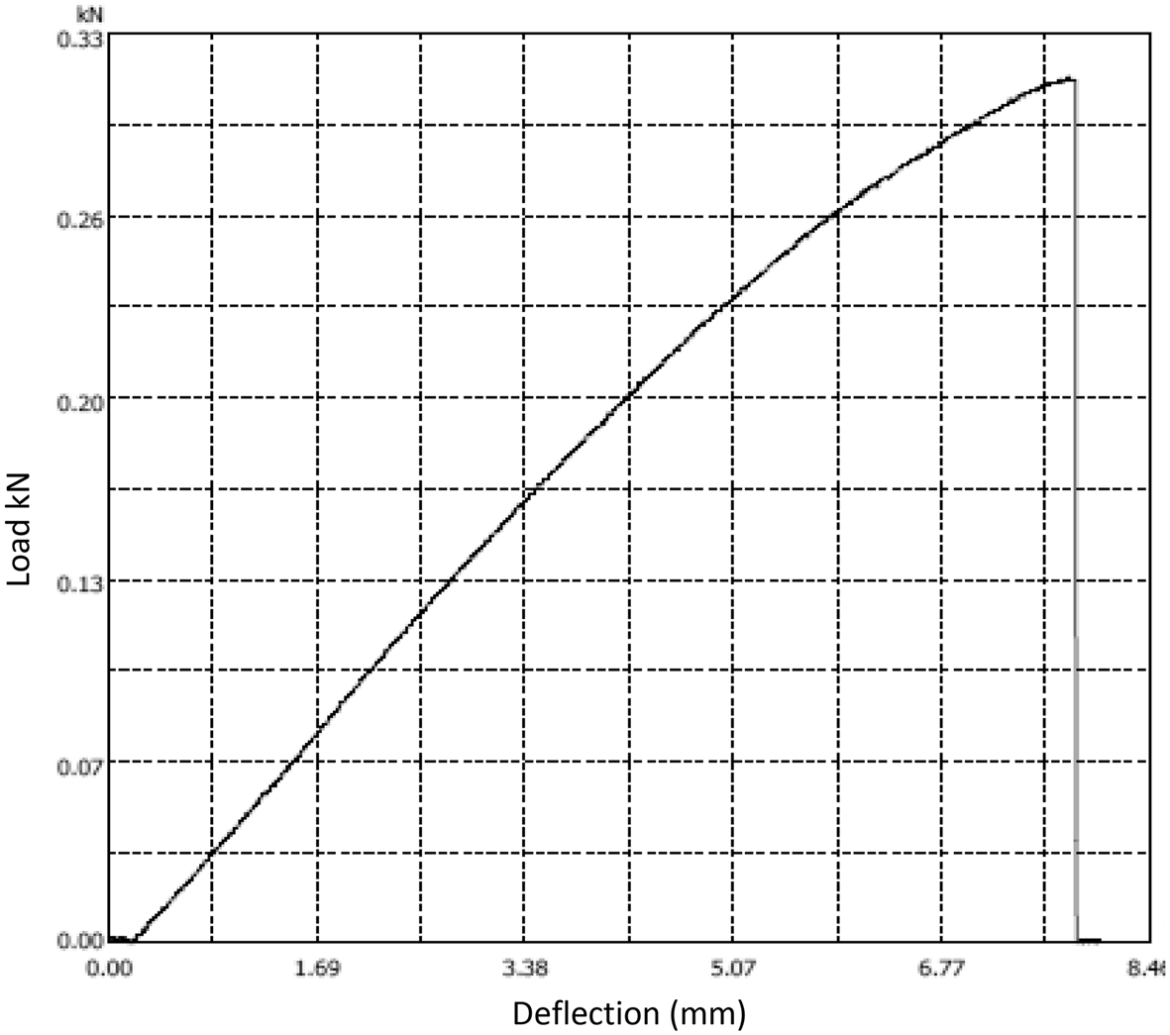
Load deflection curve for flexural test (by authors).

For modulus of rupture (MoR) determination, three samples are tested. The test is conducted using three-points bending test and the span is 150 mm. The test setup is shown in Fig. 9. The loading rate is 1mm/min for flexural strength test according to ASTM D7264M-15 (ASTM 2007). The load–deflection curve is shown in Fig. 10. The flexural strength can be calculated using Eq. (3),

3 $$MoR=\frac{3PL}{2bh*h}\times 100$$

Equation (3): Modulus of rupture equation.

The specimen number, relevant dimensions, ultimate load, Modulus of Rapture (MoR), and the standard deviation of the results are shown in Table 4.

**Table 4:** Flexural strength test results (by authors).

Specimen Number	Dimensions	Ultimate load (kN)	Modulus of Rapture (MPa)	Average modulus of rapture (MPa)	Standard deviation of MoR (MPa)
1	150 × 26 × 9.25	0.3125	31.74	30	1.61
2	150 × 26 × 9.2	0.2775	28.29		
3	150 × 26 × 9.25	0.2475	29.4		

### Visual inspection

Upon visual inspection of the surface properties of the generated panel board, it is determined that the colourless nature of the epoxy resin forming the matrix, provides the board with an authentic natural outlook Fig. 3. Thus, the natural fibers of the SCB are apparent and add aesthetic value to the board if compared to other boards in the market. This property also makes it possible to add color pigmentation to the epoxy, thus providing the developed material with a variety of color hues that would be useful for the application of the board for interior design. Table 5 presents two other similar boards that are under commercial use and some interior design shots that employ similar boards^47,48^, together with the developed board, to show where it stands in terms of appearance.

**Table 5:**
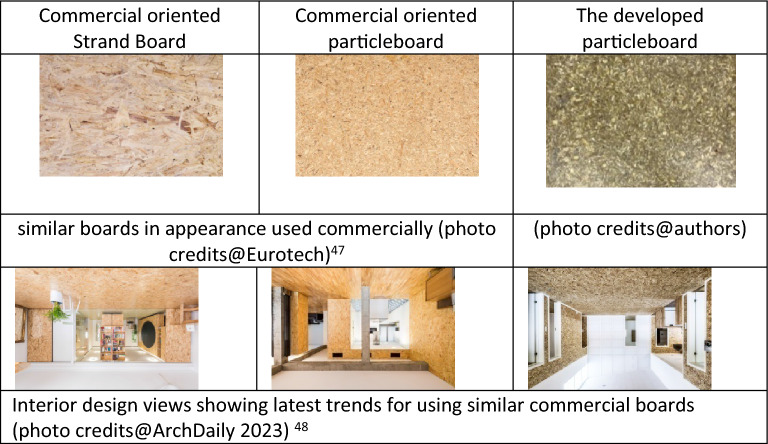
Visual appearance of developed board versus commercially used similar boards and interiors (by authors).

## Discussion

Relevant physical and mechanical properties for various similar organic boards; either particleboards, medium density fiberboards or high density fiberboards, are collected from the literature to evaluate the developed board comparatively. This comparison is shown in Table 6. To take into account the unit weight of the developed board and the mechanical properties, and to conduct a fair comparison, the different materials should be compared with respect to specific strength and specific elasticity where,

**Table 6:** Physical and mechanical properties of organicboards used in different architectural purposes in construction (By Author).

Reference	fiber material	Matrix material	Fiber volume fraction %	Density (gm/cm^3^)	Thermal conductivity W/(m k)	Water absorption (%)	MoR (MPa)	Tensile strength (MPa)	MoE (GPa)	Specific MoR (MPa cm^3^/gm)	Specific Tensile strength (MPa cm^3^/gm)	Specific stiffness (GPa cm^3^/gm)
Authors	SCB	Epoxy	39	1.08	0.093	5.16	31.7	12	1.27	29.35	11.11	1.18
^49^	Coconut Husk	no binder	1	0.250	0.046	NA	0.12	NA	NA	0.48	NA	NA
				0.350	0.068		0.68		0.088	1.94		0.25
				0.450	NA		1.94		0.368	4.31		0.817
^49^	SCB insulation board	no binder	1	0.250	0.049	NA	0.43	NA	0.102	1.72	NA	0.408
				0.350	0.055		1.51		0.392	4.31		1.12
				0.450	NA		4.16		0.957	9.24		2.127
^6^	Date-palm	Polyvinyl alcohol (PVA) to bound fibers	NA	0.203–0.245	0.038–0.051	NA	NA	6.9–10	3.8–5.98	NA	33.99–24.4	18.719–24.4
^8^	SCB	Starch	62.5	0.113	0.0520	NA	2.76	NA	NA	24.42	NA	NA
^9^	SCB and bamboo charcoal	Polyurethane	35	NA	0.130	NA	72	3.4	NA	NA	NA	NA
^10^	Pinus Elliottii Wood	Polyurethane Resin Derived from Castor Oil	94	0.75	NA	NA	36	1.6	NA	38.3	2.133	NA
^10^ ^11^	MDF	formaldehyde resin with hardener Emulsified paraffin wax	NA	0.65	0.23	6	11	0.35	1.6	16.92	0.54	2.46
	HDF		NA	0.95*	0.14**	NA	11**	19**	3.98**	24.21	20	4.21

*^50^.

**^51^.

Specific strength in tension = TS/density.

Specific strength in flexural = MoR /density.

Specific stiffness = MoE/density.

By comparing the different composites in Table 6, the developed board possess 11.11 MPa cm^3^/gm, 29.35 MPa cm^3^/gm, 1.18 GPa cm^3^/gm for specific tensile strength, specific flexural strength, and specific stiffness respectively. The relatively high density of the board can be tolerated when considering the high strength they possess. These properties reveal the potential for this board to compete with the MDF and HDF where these two products depend on the depleting natural resources of wood.

Moreover, the developed board enjoys a relatively low thermal conductivity when compared to other natural fiberboards taking into account the good mechanical properties and the aesthetically pleasing form as well. The potential of wide range of applications of this board is obvious.

The fiberboard offers authentic, natural and stylish designs that are durable and easy to maintain. Hence, one of the prospective applications can be cladding panels or tiles. In addition to the low thermal conductivity property that makes it an interior cladding board with good insulation properties.

Additionally, by examining these properties, it is determined that another potential application for utilizing such green boards is furnishing units such as tables, closets, or chairs.

## Conclusion and recommendations

The developed interior cladding panel board can be described as green and sustainable in terms of using a natural green fiber as a base material of a an abundant agro-waste material of SCB with its biodegradability, ease of accessibility, and non-toxicity properties. Sugarcane bagasse particleboard that has been produced demonstrated physical and mechanical qualities that can compete with MDF, such as thermal conductivity, modulus of rupture, and modulus of elasticity. These enhanced properties open a wide range of applications in the construction, architectural, and industrial fields. It is recommended to replicate the experiment with another moulding technology such as vacuum infusion; this may result in lower densities which would provide lower thermal conductivities to enhance this property.

This board addresses many of the SDGs such as Good Health and Well-being, Sustainable Cities and Communities, in addition to Climate Action^52^. However, to enhance the ecological properties of the board, it is strongly recommended to replicate the experiment in further research by using another matrix as binding material with better biodegradability, eco-toxicity properties to have an improved overall environmental performance, in addition to a more economic value. It is also recommended to conduct further research on how to make this industry of manufacturing boards a green industry.

From the architectural perspective, the developed board offered an aesthetical appearance and possibilities for variations through the coloring options. The visual esthetical properties of the board can be investigated by adding color pigments to the matrix as an option, or working on different particle size of the SCB to obtain a different outlook as another alternative. Additionally, developing and testing the installation methods of the board by using fixation systems that can be mechanical or chemical needs investigation. It is recommended to enhance and explore possibilities in future research on the appearance to explore possibilities of uses within the field of interior design. The u-value of each combination of layers can be calculated for the resultant wall construction upon using it as a cladding board. More properties, such as the void ratio, surface properties, and reaction with chemicals, need further investigation to determine the applicability of using it in other interior design applications, such as floor tiles and furnishing materials.

Finally, the most important aspect of using this local agro-waste is that it addresses sustainability pillars. From the environmental aspect, it offers energy savings by reducing cooling loads as it displayed good thermal insulation characteristics, thus provides thermal comfort. It is an opportunity for waste recycling, and waste management by avoiding negative environmental impact due to burning. From an economic standpoint, SCB is an abundant cheap material with a consistent supply. From the social aspect, the authentic natural appearance perhaps provides a vernacular, yet modernized outlook, in addition, to well-being through human comfort.

## Data Availability

All data generated or analysed during this study are included in this published article.

## Abbreviations

SDGs: Sustainable development goals
SCB: Sugarcane bagasse
